# Performance of Dutch Children on the Bayley III: A Comparison Study of US and Dutch Norms

**DOI:** 10.1371/journal.pone.0132871

**Published:** 2015-08-12

**Authors:** Leonie J. P. Steenis, Marjolein Verhoeven, Dave J. Hessen, Anneloes L. van Baar

**Affiliations:** 1 Child and Adolescent Studies, Utrecht University, Utrecht, The Netherlands; 2 Methods and Statistics, Utrecht University, Utrecht, The Netherlands; University of Leuven, BELGIUM

## Abstract

**Background:**

The Bayley Scales of Infant and Toddler Development-third edition (Bayley-III) are frequently used to assess early child development worldwide. However, the original standardization only included US children, and it is still unclear whether or not these norms are adequate for use in other populations. Recently, norms for the Dutch version of the Bayley-III (The Bayley-III-NL) were made. Scores based on Dutch and US norms were compared to study the need for population-specific norms.

**Methods:**

Scaled scores based on Dutch and US norms were compared for 1912 children between 14 days and 42 months 14 days. Next, the proportions of children scoring < 1-SD and < -2 SD based on the two norms were compared, to identify over- or under-referral for developmental delay resulting from non-population-based norms.

**Results:**

Scaled scores based on Dutch norms fluctuated around values based on US norms on all subtests. The extent of the deviations differed across ages and subtests. Differences in means were significant across all five subtests (p < .01) with small to large effect sizes (*η*_*p*_^2^) ranging from .03 to .26). Using the US instead of Dutch norms resulted in over-referral regarding gross motor skills, and under-referral regarding cognitive, receptive communication, expressive communication, and fine motor skills.

**Conclusions:**

The Dutch norms differ from the US norms for all subtests and these differences are clinically relevant. Population specific norms are needed to identify children with low scores for referral and intervention, and to facilitate international comparisons of population data.

## Introduction

The Bayley Scales of Infant and Toddler Development-third edition (Bayley-III) [1] are frequently used to assess cognitive, motor, and language development in infants and toddlers. The Bayley-III was developed and normed for children of the US population. However, the test and its norms are used worldwide in health care settings, as well as for scientific research purposes. In view of all factors that influence child development, such as biological characteristics, parenting, cultural habits, environmental characteristics and the interaction between these factors [2], it seems doubtful that US norms would be adequate in populations with different characteristics and languages, leading to inaccuracies in identification of referral thresholds. Overestimation of the developmental level of a child would result in non-referral of children in need of treatment and opportunities for early intervention would be missed, whereas underestimation of the developmental level would result in unnecessary referrals with increasing healthcare costs and parental concern.

Several studies on the Bayley scales have indicated that the US norms indeed may not be adequate for use in other populations. In the previous version of the Bayley scales, the BSID-II [3], US norms underestimated the developmental level of Dutch children: When using Dutch instead of US norms, preterm born children at 6 and 12 months corrected age scored significantly higher on mental and motor development [4]. Such results were also found for the Bayley-III in other countries when comparing children’s mean standardized scores to the normative mean for different age groups. For example, mean scores on cognition and receptive communication were higher than the normative US-mean in 1-year-old and 3-year old Australian children [5,6] and 6 to 24 month old Taiwanese children [7]. However, regarding expressive communication, 6 to 24 month old Taiwanese children [7], 4 to 13 month old Danish children [8] and 1-year-old Australian children [6], scored lower than the normative mean, whereas 3-year-old Australian children had higher scores compared to the normative mean [5]. These studies illustrate that US norms might not be suitable for all populations: Dependent on country, age and subtest, children in other countries sometimes develop slower, similar or quicker than children in the US.

Previous studies on norm comparisons mainly focused on specific age groups and groups that were not representative of the studied population. Also these studies did not use population specific norms. Recently a Dutch version of the Bayley-III, the Bayley-III-NL was developed and standardized for the Dutch population [9,10,11]. As Dutch norms have already been created, it is now possible to compare the US norm scores with these Dutch norm scores for the whole age-range of the Bayley-III-NL. In a large stratified sample of children in the age range from two weeks to 42 months and 15 days, we studied to what extent outcomes regarding the development of cognition, motor skills and communication differ between US norms and Dutch norms.

## Method

### Participants

Children between 2 weeks and 3.5 years old were recruited by flyers, advertisements in local newspapers and via personal connections. Parents could actively consent to participate via internet, telephone or mail. Due to this recruitment procedure, no response rate could be calculated. Children met the inclusion criteria when according to their parents, they had no physical or mental health issues, did not use medicine regularly, had a birth weight of at least 2500 grams, and a gestational age of at least 37 weeks. In addition, like the US sample, 10% of the Dutch norm sample consisted of children at risk for, or showing developmental delay. Children met the criteria for this group if they had special needs (e.g., clinical indications for low vision of motor functioning), or when parents reported presence of risk factors for development (e.g. prematurity, Down Syndrome).

To ensure representativeness for the Dutch population, the composition of the norm sample was guided by the four background demographics that also were used for the compilation of the US sample: child’s gender, parental education, parental ethnicity and geographical region. The target percentages regarding these demographics are presented in Table 1. These are based on population characteristics as provided by the Dutch Central Bureau of Statistics(CBS) [12] The categories used by the CBS to distinguish educational levels, differences in ethnicity and geographical region, fit the Dutch system and therefore these were also used for the norm sample and in this study. To optimize the representativeness of the sample, the norm sample was also weighted per age-group in order to reach the target percentages as described in Table 1, in terms of gender, geographical living region, parental ethnicity, and mother’s educational level. The weighting values were between zero and two, which is in concordance with the guidelines of the Commissie Testaangelegenheden Nederland (COTAN)[13].

**Table 1:** Background characteristics of the sample.

		Total	Population Characteristics**
Total N		1912	-
Mean age (SD)		17.8 (12.2)	-
Boys %		51.5	51.0
Mean gestational age in weeks (SD)		39.4 (2.1)	-
Mean birth weight (g) (SD)		3456.5 (608.8)	-
*Health*			
	Healthy	87.4	90.0
	Moderately preterm (%)	5.6	6.0
	Extremely preterm (%)	.9	1.0
	Down syndrome	1.4	.2
	Other (%)	4.7	2.8
*Ethnicity parents*			
	Dutch (%)	79.7	75.0
	Non-Dutch	20.3	25.0
*Educational level mother* *			
	Low (%)	13.6	16.0
	Medium (%)	37.6	39.0
	High (%)	48.8	45.0
*Living region in the Netherlands*			
	North (%)	11.7	10.0
	East (%)	22.6	21.0
	South (%)	23.6	22.0
	West (%)	42.1	47.0
*n per age group*			
A: 16 days-1 month 15 days		79	
B: 1 months 16 days-2 months 15 days		73	
C: 2 months 16 days-3 months 15 days		69	
D: 3 months 16 days-4 months 15 days		73	
E: 4 months 16 days-5 months 15 days		72	
F: 5 months 16 days-6 months 15 days		71	
G: 6 months 16 days-8 months 30 days		152	
H: 9 months 0 days-10 months 30 days		120	
I: 11 months 0 days-13 months 15 days		107	
J: 13 months 16 days-16 months 15 days		145	
K: 16 months 16 days-19 months 15 days		147	
L: 19 months 16 days-22 months 15 days		105	
M: 22 months 16 days-25 months 15 days		101	
N: 25 months 16 days-28 months 15 days		97	
O: 28 months 16 days-32 months 30 days		174	
P: 33 months 30 days-38 months 30 days		222	
Q: 39 months 30 days-42 months 15 days		105	

*’ ‘Low educational level’ refers to special education, primary school, or pre-vocational secondary education (< 12 years); ‘medium educational level’ refers to senior general secondary education, pre-university education, or secondary vocational education (13–16 years); ‘high educational level refers to higher professional education or university (17+ years).

** Refers to the percentages in the Dutch population based on information provided by the Central Bureau of Statistics (CBS; [12]).

For the current study, we included children for whom results were available for all subtests of the Bayley-III. This concerns 1912 (97.9%) of the children in the norm sample. Children at risk or showing developmental delay were included (12.6%). In Table 1, characteristics of the participants are presented for different age groups. The sample was considered to be representative for the Dutch population as proportions of children in relation to the background characteristics did not deviate more than 5 percent from the target percentages.

### Measurements

#### Background questionnaire

Mothers completed a background questionnaire containing 26 questions about family background and child-characteristics, such as date of birth, health of the child, ethnicity of children and parents, and family composition.

#### Bayley-III-NL

The Bayley-III-NL is the translated and slightly adapted version of the Bayley Scales of Infant and Toddler Development-third version (Bayley-III; [1,9,10,11]). It is an individually administered instrument that measures the developmental level of children between 16 days to 42 months and 15 days old. For administration purposes, this age range is divided into 17 age groups, just as in the original US version. The Dutch version consists of five subtests: Cognition (91 items), Receptive Communication (49 items), Expressive Communication (46 items), Fine Motor (66 items), and Gross Motor (72 items).

In the Dutch version a few adaptations were necessary in order to fit the Dutch culture and specifically the Dutch language. Changes were kept to a minimum to maintain international comparability of the Bayley-III-NL. First, five pictures in the material were adapted to Dutch culture: one in the Cognitive Scale, three in the Receptive Communication Subtest, and one in the Expressive Communication Subtest). For example an American football was changed into a soccer ball which is more common in the Netherlands. Second, two items of the Expressive Communication subtest were deleted as these do not fit Dutch language development: item “Uses Verb + *ing*” and item “Uses present progressive form” [10,11]. Third, as in the pilot study Dutch children showed a slower development in Gross Motor skills compared to the US children, the starting point for this subtest was set one age group younger than in the US version. In addition, the reversal rule was made stricter; the first five instead of three items had to be scored positively or else the items of a younger age group had to be assessed [11].

The Bayley-III-NL norms were constructed by means of continuous norming techniques using the weighted sample [9]. The construction of the Dutch norms was based on age calculated in days, as this most precisely seems to reflect the development of young children [9]; for the US norms, age-groups varying from two weeks to three months were used [1].

Like in the original US version, the standardized scores of the subtests of the Bayley-III-NL range from 1 to 19 with a mean of 10 and a standard deviation of 3. The Bayley-III-NL is a reliable and valid instrument with good psychometric characteristics in Dutch children. The reliabilities of the five subtests were assessed using Guttman’s Lambda 2 and varied from .82 to .92 [2].

### Procedure

Examiners were experienced clinicians or pedagogy students in the final year of their bachelor- or master education and all were trained to be reliable in their test administration. All examiners also scored a Bayley-III-NL assessment on film and had to acquire an inter-rater reliability level with a minimal consensus rate of 80% per subtest to pass their training. Their scores were compared to the scores of the trainer and the average kappa for all administered items over all subtests was .77 (*SD* = .05).

Two weeks prior to the planned Bayley-III-NL assessment, mothers received questionnaires and an informed consent form by mail which they were asked to complete at home. During the visit for the Bayley-III-NL assessment, the questionnaires and informed consent form were collected. Next, a trained test leader administered the Bayley-III-NL in presence of a maximum of two primary care-givers. Locations within an acceptable travel distance for the parents and their child were selected, and the rooms were free from distracting stimuli. Because young infants are only awake and alert for small periods of time and traveling to a lab could be too fatiguing, children up to six months of age were tested at home. Dependent on the age of the child and the preference of the caregiver(s) and the child, the child sat at the lap of one of the caregivers, or independently on a chair during the parts of the assessment for which sitting at a table was required. The Utrecht University Medical Center’s Medical Ethical Committee approved this study.

### Data analysis

Differences between the scaled scores based on Dutch and US norms were calculated for all children on all subtests. A one sample Multivariate analysis of variance (MANOVA) was used to test whether the mean difference scores over all subtests for the sample as a whole were equal to zero or not, and to control for inflation of type 1 error. When this MANOVA indicated significant differences between the scaled scores based on Dutch and US norms, we referred to the univariate results to see for which subtest these significant differences were found. As the mean differences might be age dependent, the same MANOVA including all five subtests was performed in the next step for each age group separately. Effect sizes (*η*_*p*_^*2*^ *)* of these results were evaluated and interpreted according to Cohen [14] with .06 or less indicating a small effect, .07-.13 a medium effect, and .14 or higher a large effect size. Finally, the proportions of children with low scores, scoring <-1 SD (i.e. a scaled scores <7) and <-2 SD (i.e. a scaled score <4) based on Dutch norms and US norms, were compared by means of McNemar analyses. Analyses were performed using SPSS 20.0.

## Results

**Fig 1:**
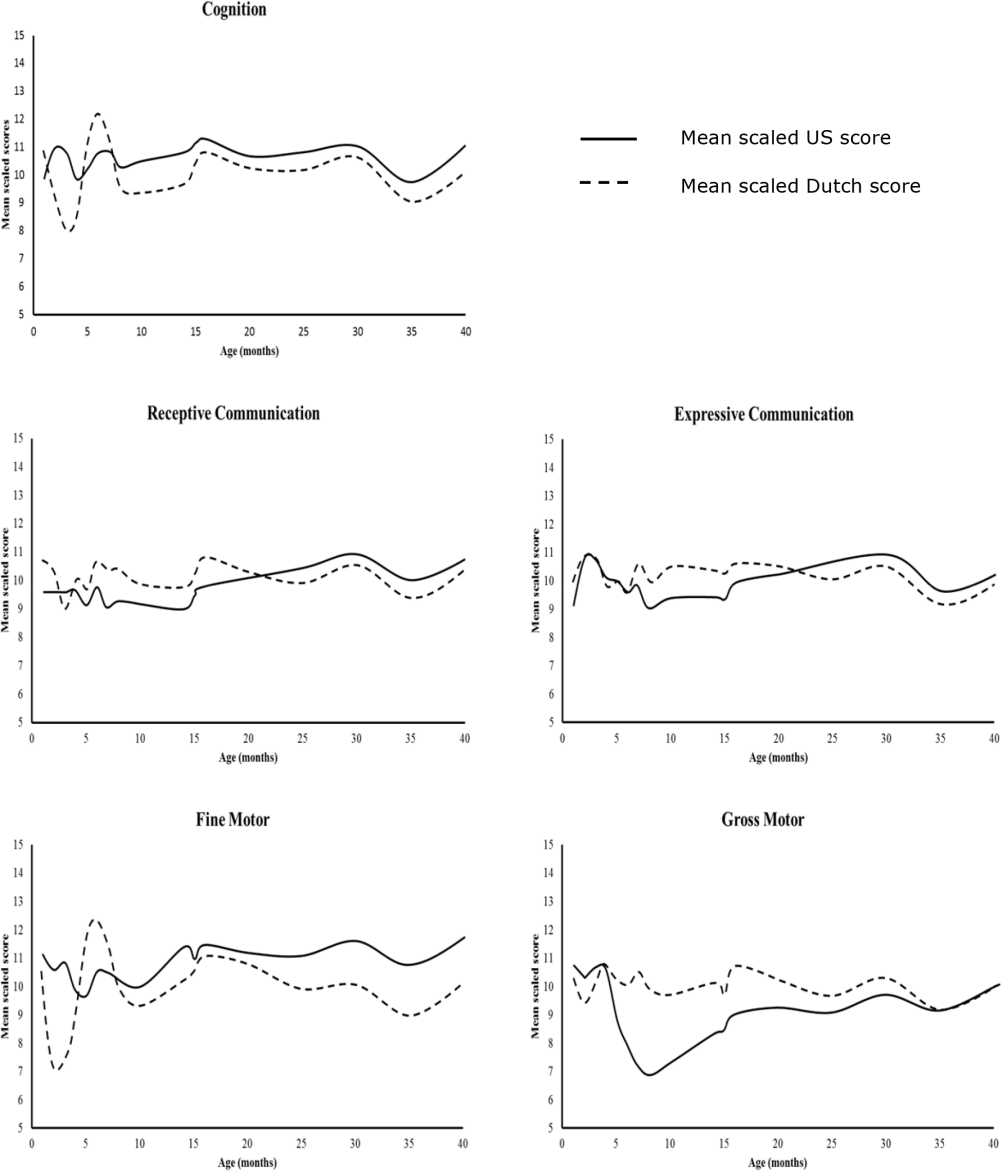
Average US and Dutch scaled scores in relation to age.

The graphs in Fig 1 display the average scaled scores of each age group per subtest, based on Dutch norms (dotted line) and US norms (continuous line). The graphs show that the Dutch and the US scores deviated from each other and that these deviations differed across age groups and subtests.

Results of the overall MANOVA over all age groups revealed that for all subtests, the mean differences between the scaled scores based on the Dutch and US norms significantly deviated from 0, with a large effect size, *F*(5,1907) = 449,99, *p* <. 01, *η*_*p*_^*2*^ = .54, indicating a significant difference between the Dutch norms and the US norms. Next, the univariate results (see Table 2) showed significant differences for all subtests with large effect sizes for the Cognition, Fine Motor, and Gross Motor subtests of .15, .16, and .25 respectively, and small effect sizes for the Receptive Communication and Expressive Communication subtests of .06 and .05 respectively. The mean differences presented in Table 2 also provide information on the size of the standard deviation in relation to the effect sizes.

**Table 2:** MANOVA results per subtest over all age groups.

	Mean difference	CI 95%	F	df	p	*η*_*p*_^*2*^
Cognition	-0.58	[.64,-.51]	324.22	1	< .01	.15
Receptive Communication	-0.33	[.27, .39]	120.77	1	< .01	.06
Expressive Communication	-0.25	[.19, .31]	065.80	1	< .01	.03
Fine motor	-0.82	[-1.25, .01]	357.11	1	< .01	.16
Gross motor	-1.09	[1.01, 1.17]	676.01	1	< .01	.26

Note. The Mean difference is calculated by the scaled score based on Dutch norms minus the scaled score based on US norms. Mean differences < 0 indicate that the score based on the US norms was higher than the scaled scores based on the Dutch norms. Mean differences >0 indicate that the scaled score based on the US sample is lower than the scaled score based on the Dutch sample.

The graphs in Fig 1 indicate that the extent of the deviations fluctuated across age groups for all subtests. Table 3 presents the mean difference between the scaled scores based on the Dutch and the US norms per age group for all subtests. The smallest mean difference of .01 was found for Expressive Communication for age group B (1 months 16 days-2 months 15 days). The largest mean difference of 3.18 was found for Gross Motor skills for age group G (6 months 16 days-8 months 30 days.), which equals more than 1 SD based on the Bayley-III-NL scaled scores.

**Table 3:** Mean differences and confidence intervals per Bayley-III-NL age group.

	Cognition		Receptive Communication		Expressive Communication		Fine Motor Skill		Gross Motor Skills	
Age group	Mean difference (SD)	CI 95%	Mean difference (SD)	CI 95%	Mean difference (SD	CI 95%	Mean difference (SD	CI 95%	Mean difference (SD)	CI 95%
A	.95 (1.82)	[.54, 1.36]	1.09 (2.09)	[.62, 1.56]	.73 (1.47)	[.40, 1.06]	-.62 (2.81)	[-1.25, .01]	-.43 (1.72)	[-.82,-.05]
B	-1.85 (.83)	[-2.04, -1.66]	.08 (1.05)	[-,16, .33]	.01 (1.23)	[-.27, .30]	-3.36 (1.57)	[-3.72, -2.99]	-.86 (1.59)	[-1.24,-.49]
C	-2.68 (.63)	[-2.83, -2.53]	-.46 [.95)	[-.69,-.24]	-.10 (.62)	[-.25, .05]	-3.15 (1.26)	[-3.45, -2.84]	-.51 (.93)	[-.73,-.28]
D	-1.38 (.89)	[-1.59, -1.18]	.27 (.71)	[.11, .44]	.29 (.81)	[-.48,-.10	-1.04 (1.20)	[-1.32,-.76]	.15 (1.37)	[-.17, .47]
E	.833 (1.11)	[.57, 1.10]	.56 (1.03)	[.31, .80]	.04 (1.16)	[-.23, .31]	2.01 (1.39)	[1.69, 2.34]	1.46 (1.11)	[1.20, 1.72]
F	1.31 (1.10)	[1.05, 1.57]	.92 (1.13)	[.65, 1.18]	.01 (1.44)	[-.33, .36]	1.68 (1.88)	[1.23, 2.12]	1.94 (1.87)	[1.50, 2.39]
G	.33 (1.07)	[.16, .50]	1.25 (1.08)	[1.08, 1.42]	.74 (1.51)	[.49, .98]	.78 (1.39)	[.55, 1.00]	3.18 (1.16)	[2.99, 3.36]
H	-.70 (.97)	[-.88,-.53]	1.08 (.59)	[.98, 1.19]	.78 (1.04)	[.60, .97]	.04 (.78)	[-.10, .18]	3.01 (1.09)	[2.81, 3.21)
I	-1.13 (1.07)	[-1.34,-.93]	.68 (.80)	[.53, .84]	1.10 (1.41)	[.83, .1.37]	-.67 (.82)	[-.83,-.52]	2.32 (1.55)	[2.02, 2.61]
J	-1.05 (1.12)	[-1.23,-.86]	.76 (1.31)	[.54, .97]	.86 (.92)	[.71, 1.01]	-1.28 (.98)	[-1.44, -1.12]	1.77 (.99)	[1.61,1.94]
K	-.72 (1.57)	[-.98,-.47]	.76 (1.36)	[.53, .98]	.80 (1.67)	[.53, 1.07]	-.57 (2.08)	[-.91,-.23]	1.27 (1.54)	[1.02, 1.52]
L	-.52 (1.07)	[.-73,.-32]	.92 (1.07)	[.72, 1.13]	.70 (.82)	[.54, .85]	-.48 (.76)	[-.62,-.33]	1.64 (1.09)	[1.43, 1.85]
M	-.48 (.94)	[-.66,-.29]	.23 (1.24)	[-.02, .47]	.34 (.67)	[.21, .47]	-.46 (1.09)	[-.67,-.24]	.99 (1.83)	[.63, 1.35]
N	-.66 (.84)	[-.83,-.49]	-.51 (.99)	[-.71,-.31]	-.19 (.70)	[-.33,-.05]	-1.17 (1.12)	[-1.39,-.94]	.53 (1.18)	[.29, .76]
O	-.36 (1.05)	[-.52,-.20]	-.36 (1.05)	[-.51,-.20]	-.41 (.87)	[-.54,-.28]	-1.51 (1.32)	[-1.70, -1.31]	.58 (1.60)	[.34, .82]
P	-.73 (1.23)	[-.89,-.57]	-.61 (1.02)	[-.75,-.48]	-.50 (1.35)	[-.67,-.32]	-1.76 (1.51)	[-1.96, -1.56]	.07 (1.50)	[-.13, .27]
Q	-.95 (1.15)	[-1.17,-.73]	-.45 (1.09)	[-.66,-.24]	-.32 (1,83)	[-.68, .03]	-1.58 (2.06)	[-1.98, -1.18]	-.11 (1.64)	[-.42, .21]

Note. The Mean difference is calculated by the scaled score based on Dutch norms minus the scaled score based on US norms. Mean differences < 0 indicate that the score based on the US norms was higher than the scaled scores based on the Dutch norms. Mean differences >0 indicate that the scaled score based on the US sample is lower than the scaled score based on the Dutch sample

As Fig 1 shows that the size of the differences between the scores based on the Dutch and the US norms differed per age group, the results were also analyzed with MANOVA’s including all subtests for each Bayley-III-NL age group separately. The second column in Table 4 displays the effect sizes regarding the multivariate analyses in which all five subtests were included. Large effect sizes were found for the differences between the scaled scores based on the US and Dutch norms for all age groups, but not consistently for specific subtests or for specific age groups (Table 4). For Cognition, effect sizes were generally large for all age groups. For the Receptive Communication subtest, effect sizes were generally large with the exception of four age groups. Regarding the Expressive Communication subtests, for children ≥ 6 months and 15 days most effect sizes were large, whereas small to moderate effect sizes were found for the age groups between 1 month 15 days to 6 months 15 days old, which represents the period of preverbal development. Regarding the Fine Motor subtest, differences between the US and Dutch norms were largest in children between 1 month 15 days to 8 months 30 days old. The effect sizes for the older age groups, from 9 months to 42 months and 15 months old, fluctuated from small to large. For the Gross Motor scale, most effect sizes were large for the age- groups 1 month 15 days to 28 months 15 days. For the older age groups, only moderate to small effect sizes were found. For most of these small effect sizes, 0 falls within the confidence interval, indicating that no significant difference exists between the scaled scores based on the Dutch norms and the US norms.

**Table 4:** Partial eta squared values per age group for all subtests resulting from the MANOVA analyses.

	Mulivariate (Overall)	Cognition		Receptive Communication		Expressive Communication		Fine Motor		Gross Motor	
Age—group	*η*_*p*_^*2*^	+/-	*η*_*p*_^*2*^	+/-	*η*_*p*_^*2*^	+/-	*η*_*p*_^*2*^	+/-	*η*_*p*_^*2*^	+/-	*η*_*p*_^*2*^
A	.49	+	.22	+	.22	+	.20	-	.*05*	-	.*06*
B	.90	-	.84	*+*	.*01*	*+*	.*00*	-	.82	-	.23
C	.96	-	.95	-	.20	*-*	.*03*	-	.86	-	.23
D	.82	-	.71	+	.13	+	.11	-	.44	*+*	.*01*
E	.76	+	.36	+	.23	*+*	.*00*	+	.68	+	.64
F	.71	+	.59	+	.40	*+*	.*00*	+	.45	+	.52
G	.89	+	.09	+	.57	+	.19	+	.24	+	.88
H	.93	-	.34	+	.77	+	.37	*+*	.*00*	+	.89
I	.90	-	.53	+	.43	+	.38	-	.40	+	.69
J	.93	-	.47	+	.25	+	.47	-	.63	+	.76
K	.73	-	.18	+	.24	+	.19	-	.07	+	.41
L	.81	-	.20	+	.43	+	.42	-	.28	+	.69
M	.60	-	.20	*+*	.*03*	+	.21	-	.15	+	.23
N	.65	-	.38	-	.21	*-*	.*07*	-	.52	-	.17
O	.67	-	.11	-	.10	-	.19	-	.57	-	.12
P	.61	-	.26	-	.27	-	.12	-	.58	*-*	.*00*
Q	.52	-	.41	-	.15	*-*	.*03*	-	.37	*-*	.*00*

Note. Effect sizes are all statistically significant, p < .01, except those in italics. Directions of the effects are indicated for each subtest. A + indicates that the scores based on the Dutch norms > scores based on the US norms. A–indicates that that the scores based on the Dutch norms <scores based on the US norms

Using a scaled score of 7 as a cutoff point, McNemar analyses showed that for all subtests, except Receptive Communication, significantly different proportions of children with low scores were found using Dutch and US norms (Table 5). When using the US norms instead of the Dutch norms, fewer children scored below 1 or 2 SD in Cognition, Fine Motor and Expressive Communication and more children regarding Gross Motor functioning. Regarding the Receptive Communication subtest, a similar proportion of children scoring below 1 SD, but less children scoring below 2 SD were identified when using the US norms. In addition, McNemar analyses have been performed for 4 age groups (see Table 5). For all age groups, the proportions of children scoring below 1SD using Dutch and US norms differed significantly for most subtests. The difference between the proportions of children who scored below 2 SD using Dutch and US norms, was significant for only few subtests for the youngest three age groups and for most subtests for the oldest age group. In general, more children scored below 1 and 2 SD using the Dutch norms in comparison to using the US norms.

**Table 5:** Proportion of children with low scores based on US or Dutch norms.

	US norms	Dutch norms	US norms	Dutch norms
	< 1SD %	< 1SD %	< 2SD %	< 2SD %
*All age groups*				
Cognition	05.3	13.0**	1.5	3.1**
Receptive Communication	12.1	12.1**	2.0	2.8**
Expressive Communication	09.7	12.3**	1.9	2.8**
Fine Motor	05.6	14.1**	1.2	3.3**
Gross Motor	19.7	13.9**	5.9	2.8**
*Age groups*				
*0–6months 15 days*				
Cognition	6.6	14.2**	.9	1.8**
Receptive Communication	11.0	11.2**	0	2.7**
Expressive Communication	6.7	12.2**	.5	1.4**
Fine Motor	6.6	26.1**	.7	4.8**
Gross Motor	8.5	11.7**	.9	1.8**
*6 months 16 day -13 months 15 days*				
Cognition	2.6	11.3**	.3	1.8**
Receptive Communication	15.8	7.4**	1.1	0.3**
Expressive Communication	8.2	11.6**	.8	.3**
Fine Motor	2.9	2.1**	0	.3**
Gross Motor	43.4	15.1**	12.7	1.3**
*13 months 16 day -25 months 15 days*				
Cognition	3.8	12.4**	.6	1.6**
Receptive Communication	15.1	14.5**	2.2	2.0**
Expressive Communication	11.9	8.2**	1.6	.6**
Fine Motor	4.0	4.0**	1.0	.4**
Gross Motor	18.3	10.8**	4.0	1.4**
*25 months 16 day -42 months 15 days*				
Cognition	7.4	13.7**	3.5	6.2**
Receptive Communication	8.0	13.7**	3.8	5.2**
Expressive Communication	11.0	16.1**	3.8	6.3**
Fine Motor	7.9	21.2**	4.5	7.4**
Gross Motor	14.0	17.2**	6.7	5.7**

Note. Scaled scores of < 1SD correspond to scaled scores <7 indicating a low score which may reflect a developmental delay in the subtest domain and scaled scores of < 2SD correspond to scaled scores <4, which may indicate a severe delay in the domain examined.“

*p < .05;

** p < .01

## Discussion

For a large group of Dutch children, significant differences were found between their scores based on the Dutch norms and their scores based on the US norms, on all subtests of the Bayley-III-NL. Overall, effect sizes of the differences between the scores based on the Dutch and US norms were large. Analyses concerning the proportions of children with low scores, that may indicate a developmental delay (i.e., below 1 SD and 2 SD), showed that this concerns clinically important differences. Regarding the Cognition, Fine Motor, and Expressive Communication subtests, under-referral might have resulted from the US norms, as fewer children would have been identified with a developmental delay compared to the Dutch norms. The reverse was found for the Gross Motor subtest: The use of US-norms would have resulted in over-referral, as more children would have been identified with a developmental delay compared to the Dutch norms. These results regarding over- and under referral are to some extent age-dependent. The largest difference between Dutch and US scores was found for the Gross motor subtest: For children of approximately nine months old, the difference was one standard deviation, and the mean of the Dutch children resembled that of seven months old US children. These findings illustrate important differences in functioning and developmental levels of children in two western populations. These results are in accordance with earlier studies from different countries that compared the Bayley results for children to the US norms [4,5,6,7,8].

In relation to the findings of this study, it is important to realize that some adaptations were needed to make the Bayley-III appropriate for the Dutch population. Besides translation, some changes were made to the Communication subtests in accordance with Dutch culture which is described earlier. A previous study evaluated whether the original item sequence of the Bayley-III-in which the items increase in difficulty- would be adequate for assessment of the development of Dutch children, and it was concluded that the same item sequence could be applied [11]. Thus, also for the adapted items the level of difficulty was adequate in relation to the pattern of increasing difficulty of the items which indicates that the adaptation to the items did not result in too easy or too difficult items for the children in relation to age. It is therefore unlikely that the changes caused the differences between the scores based on the Dutch and the US norms. Furthermore, only small to moderate differences between Dutch and US norm scores were found for the oldest age group on the Expressive Communication subtest in which two items were deleted. In addition, another adaptation was made to the starting point and reversal rule of the Gross Motor subtest, as described under Methods. For some children, this could have resulted in the administration of more items, and accordingly more mistakes made by these children. As a result, the use of US norms could have led to lower scores at all ages in comparison to the Dutch norms. However, for several age groups the Dutch norms were higher or comparable to the US norms. Therefore, it seems unlikely that the adaptations that needed to be made for the Dutch version of the Bayley scales solely explain the differences between the US and Dutch norm scores.

An important explanation for the differences between the norm scores concerns the constellation of the Dutch and US population that underlies the samples used for the norm construction. For the development of the Bayley-III-NL and its norms, the norming and validation procedure as used in the US was replicated. However, due to cultural differences and differences between the constellation of the populations, a perfect replication was not always possible. Both norming samples had a constellation representative for the population based on the same background characteristics concerning gender, parental education, ethnicity, and geographical region. The categories used to distinguish educational levels, differences in ethnicity and geographical region in the Netherlands were based upon the distinctions used by the CBS [12] and therewith fit the Dutch system. However, concerning ethnicity in the US, 40% of the population was from a White background, 14% African American, 20% Hispanic, 4% Asian and 1% was coded as Other [1,15]. For the Dutch population different categories were used: 75% originally Dutch (White Caucasian) versus 25% non-Dutch parents [12]. Previous studies showed that developmental trajectories of children with different ethnic backgrounds, even within the same country, were significantly different for motor skills [16] and language skills [17]. Therefore, the difference in constellation of the Dutch and US norming sample regarding ethnicity of the parents might have contributed to the differences between the norms.

Another important factor related to developmental outcome is maternal educational level. In the US, educational level was measured in years and 42% of the parents had a low-, 30% a medium- and 28% a high level of education [15]. In the Netherlands, 16% of mothers between 25 and 45 years of age had a low-, 40% a medium- and 44% a high level of education [12]. For the Bayley-III-NL, analyses regarding the association between mother’s educational level and the scaled scores of the Dutch norm sample revealed significant differences between the scaled scores of children of mothers with a low, medium and high education, with increasing age: Children of highly educated mothers generally had higher scores on the subtests Cognition and Receptive Communication compared to children of lower educated mothers [9]. This is in concordance with earlier studies, which showed that children of parents with a lower SES, including a lower educational level, had poorer language skills in comparison to children of parents with a higher SES and poorer executive functioning skills [18]. Thus the difference in constellation of the Dutch and US norming sample regarding educational levels might also have contributed to the differences between the norms.

For the youngest age groups, between 15 days and 10 months of a age, a swing in the scores based on the Dutch norms was found regarding Cognition and Fine Motor Skills. This might be explained by the fact that the norms of the Bayley-III-NL were created based on a weighted sample and based on age calculated in days, whereas the results of this study are based on an un-weighted sample and the means are presented for age groups. However, more research is needed on the relation between this swing in scores for the young children and the Bayley-III-NL and why this is seen specifically in two of the subtests.

It is concluded that outside the US, the use of population specific norms instead of the US norms is preferable. However, it is costly and time-consuming to create such norms. When population specific norms are unavailable, a matched control group should be used of children from the same population and assessed at the same ages as the studied group. Using these matched control groups as a reference may be more reliable when norms of a country with a more similar culture and constellation of the population than that of the US, are used. When data from a matched control group is not available, using norms of a more similar country might be a better alternative than using the US norms. However, caution is still needed when interpreting the results.

## Conclusion

The current study shows the importance of population-specific norms for the interpretation of developmental test results. Therefore, in the Netherlands, the Dutch population specific norms should be used for all subtests and all ages.
